# Multiple Intelligences in Teaching and Education: Lessons Learned from Neuroscience †

**DOI:** 10.3390/jintelligence6030038

**Published:** 2018-08-31

**Authors:** Branton Shearer

**Affiliations:** Multiple Intelligences Research and Consulting, Inc., Kent, OH 44240, USA; sbranton@kent.edu

**Keywords:** multiple intelligences, culture matters, every brain is unique—activate strengths, know thyself, embodied cognition/emotional rudder, and make it mean something

## Abstract

This brief paper summarizes a mixed method review of over 500 neuroscientific reports investigating the proposition that general intelligence (*g* or IQ) and multiple intelligences (MI) can be integrated based on common and unique neural systems. Extrapolated from this interpretation are five principles that inform teaching and curriculum so that education can be strengths-based and personalized to promote academic achievement. This framework is proposed as a comprehensive model for a system of educational cognitive neuroscience that will serve the fields of neuroscience as well as educators. Five key principles identified are culture matters, every brain is unique—activate strengths, know thyself, embodied cognition/emotional rudder, and make it mean something.

## 1. Introduction

The pieces of a scientific puzzle are falling into place. For 35 years teachers, students, and parents have been stuck in the middle of the war of words among psychologists regarding the nature of human intelligence. In my view, an interpretation of the neuroscience evidence now builds a coherent bridge between general intelligence (*g* or IQ) and multiple intelligences (MI) [1]. The remainder of this article is based on a similarly personal view, which is most likely not shared by most experts. This battle among theorists has resulted in confusion and unhappy compromises as teachers struggle to serve two masters. On the one side is the IQ tradition that argues that intelligence is unitary and mainly associated with academic skills (reading, math, and such). This tradition advocates for a standardized curriculum emphasizing basic skills development. On the other side are advocates for personalized instruction based on the idea of multiple intelligences [2]. They argue that human intelligence cannot be summed up with a single number; it is more than scholastic ability; and that student learning will increase with differentiated instruction that emphasizes strength-based activities.

For 35 years a wave of teachers around the world [3] has agreed with Howard Gardner that their students display very different cognitive profiles, even among those with similar IQ scores. Teachers want to customize their instruction and curriculum accordingly but have been thwarted by public policy and institutional guidelines to quickly raise academic test scores by (for the most part) “teaching to the test”. The result? No progress. Standardized national academic test scores have remained stagnant despite more than two decades of high stakes testing regimes in all 50 states [4,5].

Other barriers to progress are the outdated and inaccurate views (however pervasive among traditional psychologists and educational administrators) that the theory of multiple intelligences is invalid and ineffective [6,7]. This arose from the misguided opinion that MI is somehow against the development of academic skills such as reading and math. Nothing could be further from the truth.

Neuroscience evidence now reveals a neural bridge between IQ-type academic skills and the eight intelligences—linguistic and logical-mathematical (most closely related to academic achievement) and interpersonal and intrapersonal (also associated with school success); and spatial, musical, kinesthetic, and naturalist. The debate of “IQ vs. MI” is based on outdated model of human intelligence. Traditions rooted in a 19th century understanding of the mind are slowly evolving to keep up with the insights provided by advances in neuroscience.

A good scientific theory accurately describes behavior and has predictive power. In 1983 Gardner made several observations about human intelligence that a wealth of neuroscience evidence accumulated over the past 35 years has confirmed. First, academic skills (and IQ) are most closely associated with the linguistic and logical-mathematical intelligences. Second, there are unique neural architectures responsible for each of the intelligences (Table 1) [1]. Third, each intelligence can be expressed in several qualitatively different ways, including analytical/practical, creative cognition, insight/intuition, and aesthetic judgment [8].

## 2. Neuroscientific Evidence Supporting the Validity of MI Theory

The main criticism of MI is that it lacks empirical, experimental evidence of its validity [6,7]. General intelligence is considered to be valid because there is a wealth of test data amassed for more than 100 years—while there are no tests to measure the eight intelligences. Unrecognized by most researchers is the sizable number of brain studies that are matched to the multiple intelligences. This is a trove of scientific data scattered among many journals that are unread and largely incomprehensible to most non-neuroscientists—until recently.

The validity of any new idea can be difficult to establish especially for a theory of intelligence that challenges prevailing ideas and does not lend itself to psychometric testing. Using a rational-empirical methodology, more than 500 studies of brain function (largely fMRI experiments) were matched to the skills and abilities integral to each of the eight intelligences. Multiple studies of the core abilities for each intelligence were included to maximize reliability.

To summarize, an initial review of more than 318 experiments found a pattern of neural activations well-aligned with the cognitive components for each intelligence [1]. This was followed by a study of 417 experiments examining specific skill units within each intelligence and their relationships to each other, the other intelligences, and general intelligence [9]. A third review of 420 reports found that there are observable and meaningful differences in the neural activation patterns among skill level ability groups in four levels of brain analysis: primary regions, subregions, particular structures, and multi-region activations [10]. A study of 48 resting-state experiments found seven to fifteen intrinsic, functionally connected neural networks that are closely associated with seven of the eight intelligences [11]. Lastly, the neural architectures cited for general intelligence were compared with a proposed new category of Cognitive Qualities associated with the multiple intelligences. This investigation of 94 neuroscientific studies demonstrated support for the coherence of three Cognitive Qualities (creative cognition, insight/intuition, and aesthetic judgment) that are valued abilities integral to the definition and practical expression of each of the eight intelligences [8].^1^

Taken together, these investigations indicate that the multiple intelligences have clear, logical, and coherent neural patterns that are comparable to those identified with general intelligence. These data lend support to the proposition that each of the eight intelligences have unique neural architectures and that the idea of general intelligence is not incompatible with MI theory.

An intelligence differs from a *skill* in its depth, range, and complexity. Each of the multiple intelligences is a composite of related skills and this accounts for their complicated neural architectures. These detailed neural analyses provide a basis for future experimental tests of their ecological validity. However, because of the social-cultural aspects of the intelligences a neural description for MI may only be a *framework* rather than a complete analysis.

## 3. Using Neuroscience to Leverage Student Success with the Multiple Intelligences

**Figure 1:**
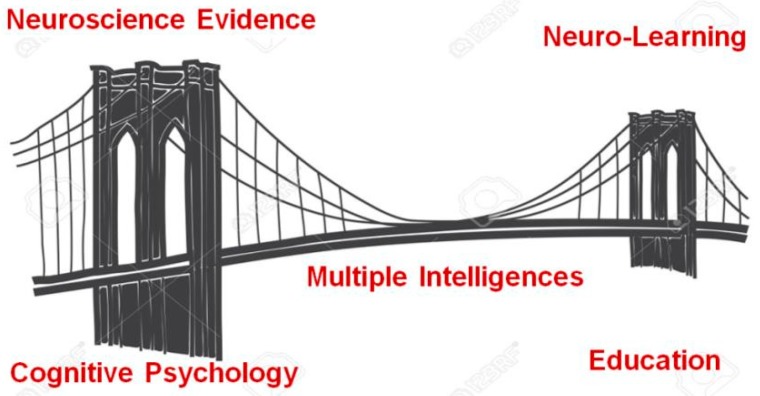
Personalized educational cognitive neuroscience: a framework. *Note*: The bridge between existing psychological models of cognition/behavior and education is spanned by multiple intelligences theory supported by neuroscience validity and efficacy evidence.

Perhaps of greater consequence are the practical implications of these scientific observations for teaching and learning. As educators worldwide were exploring diverse ways to implement MI theory, neuroscientists were giving birth to the new field of educational cognitive neuroscience to answer the question: How can insights into brain processes enhance education? Of course, the answer to this question is not simple nor obvious, in fact, John Bruer [12] famously called the distance between the neuroscience lab and the classroom a “bridge too far”. He later concluded that what is needed was advanced cognitive science theories to properly interpret the neuroscientific evidence [13]. This is where MI theory serves as a “user interface” between our neural hardware and the cognitive software that activates learning “apps” in the classroom (as well as in everyday life). See conceptual framework in Figure 1.

Each of the multiple intelligences can serve as “delivery routes” to personalize important cognitive and emotional processes underlying learning such as attention, memory, motivation, creative cognition, problem solving, and understanding [14,15,16,17]. How best to navigate these cognitive “routes”? We have neuroscience evidence to lend support to several different guiding principles. Each teacher and institution can interpret the principles and their underlying evidence according to the needs and goals of their particular situations.

Perhaps it is best to begin with a list of the most vital and vexing questions posed by teachers over the millennium.

*How to set the stage of the classroom/school to create the context for maximum learning?*
*How to enhance cognitive engagement in the instruction and curricular materials?*
*How to promote academic excellence?*
*How to teach for effective transfer of knowledge from the classroom to real life?*
*How to develop the “whole child” and instill the love of lifelong learning?*

The following descriptions of five key ideas extracted from the neuroscience literature sketch a framework that speaks to the disparate worlds of the lab and the classroom. These ideas are well supported by the evidence but are offered as an initial sketch as a kind of “communicating bridge” between cognitive scientists and teachers (Table 2).

**Key Idea 1.** Creating a Multiple Intelligences—Inspired Culture [16,17,18,19,20,21]

“…the brain and its neuronal activity must be considered a hybrid of both biological and social influences. In other words, our brains are biosocial. The brain is a relational organ that bridges the gap between the biological world of the organism and the social world of the environment and its culture”.

[18] (p. 352)

A distinct advantage of embedding MI in the learning culture is that it can easily span across diverse cultures because of its cross-cultural origins. Every school represents a cultural system of educational beliefs, social ideas, and practices. As learning culture leaders, teachers can positively frame each child’s experience by simply acknowledging that we each have our unique profile of MI history, preferences, and perspectives. The natural language of MI can be used to advantage when communicating with culturally different students and their families. The foundation is to acknowledge and value each of the multiple intelligences as important, valuable, and potentially useful to each child in the classroom.

**Key Idea 2.** Every Brain is Unique—Activate Strengths! [15,22,23,24,25,26]

“…neuroimaging studies clearly show that patterns of brain activation and structure vary in systematic ways between individuals differing in working memory and other higher cognitive abilities. Both experience and genetic factors may contribute to such individual differences… has implications for human performance”.

[22] (p. 70)

Students all have uniquely configured neural wiring that influences how they perform on classroom tasks. Teachers might experience great anxiety at the thought of having to cater to the learning profiles of so many different student brains. An impossible task! But perhaps with advances in computer software and apps and innovative assessments we are making progress towards the goal of personalization of instruction, so that students with specific strengths can exercise some choice about how information is presented to them. My own work in validating a standardized assessment—Multiple Intelligences Developmental Assessment Scales (MIDAS^®^)—shows promise as a tool to understand the cognitive and neural differences among students [23,24,25]. This is a useful tool providing a practical bridge between neuroscientists and educators seeking to understand the minds and brains of individuals.

**Key Idea 3.** Know Thyself [2,27,28,29]

“Intrapersonal intelligence involves the capacity to understand oneself, to have an effective working model of oneself—including one’s own desires, fears, and capacities—and to use such information effectively in regulating one’s own life”.

[2] (p. 43)

Neuroscience investigations into how the brain processes intrapersonal intelligence can be categorized into several distinct functions including: self-awareness, self-regulation, and executive functions. The frontal lobes and cortical midline structures (CMS) are known to be the core processing regions for many self-functions [27]. There are an unlimited number of ways that teachers can build into every subject activities to promote self-regulation and executive functions associated with excellence and achievement [28]. It begins with the teacher enhancing the students’ self-understanding and appreciation for the potential of their unique MI strengths.

**Key Idea 4.** Embodied Cognition and the Emotional Rudder [19,30,31,32]

“Recent findings in the neurosciences indicate reciprocal and parallel neural pathways between the cerebellum—traditionally viewed as controlling gross and fine motor functions but now hypothesized to play a role in thought itself—and the frontal cortex, where working memory and executive functions such as planning, monitoring, task management, and focusing attention occur”.

[19]

The relationship between the body and the mind is now recognized by neuroscientists as being bi-directional and parallel, rather than just the mind directing the body. Immordino-Yang has gone even further in detailing “a framework that situates the emotional brain and its physiological regulatory functions ecologically, spiraling from bodily behavior to embodied neural functioning to social functioning to cultural functioning” [19] (p. 360). These findings point the way forward for teachers to create opportunities for students to translate subject content into physical movements to maximize memory and understanding.

Awareness of one’s body goes beyond mere physiology associated with the maintenance of life. It is also a platform upon which emotions are played out and translated into feelings. Damasio’s “somatic marker hypothesis” cites physical responses as important elements in decision-making and judgments [30]. When we direct students’ attention to their physical and emotional responses to a topic, we are providing them with a powerful marker for that information that is accessible in their real life beyond the classroom. Making these connections may provide the keys to enhanced transfer of learning from the classroom to daily life.

**Key Idea 5.** Make it Mean Something! [19,27,29,31,33]

“…Feelings are influenced by powerful, subjective, cognitive elaborations, and cultural interpretations of bodily and mental states in context. Unlike emotions, feelings are conscious and can sometimes become reportable. Feelings contribute to self-narratives and meaning-making”.

[19] (p. 349) (emphasis added)

Mary Helen Immordino-Yang’s [19] research into self-narratives and meaning-making belies the view that facts and rational thought can be separated from feelings or practical action. Emotions and feelings are essential rudders that regulate and guide our thinking. They guide how we process new information to answer questions such as: Is this information of only temporary and limited importance? Or is it profoundly important and should I make the effort to rearrange my thinking to accommodate it?

The importance of “meaning making” to maximize engagement, learning, and cognitive transfer has been highlighted by a number of educational neuroscience researchers [27,31,33]. Such activities activate multiple neural regions and intelligences in the service of enhanced cognitive and emotional engagement.

## 4. Conclusions

Self-leadership for life-long learning is the ultimate goal for a person’s education—cultivating the knowledge that one has valuable intellectual abilities that can be developed and used to contribute meaningfully to one’s community. The multiple intelligences perspective contributes to this endeavor. Understanding how education can develop intrapersonal intelligence brings us back to the essential integration of the self within a context and a culture.

The application of neuroscience ideas in schools and classrooms is a complex endeavor and we may only be at the beginning of a long journey towards the goal of an effective interaction between neuroscientists and educators. Multiple intelligences theory provides a broad map of the software of the mind that is aligned with cognitive science and general intelligence. Cultural studies are revealing unspoken assumptions and priorities embedded in schooling that influence instruction and curriculum. The present investigation proposes that the theory of multiple intelligences provides a comprehensive framework for this array of factors influencing the design of instruction and curriculum that will be strengths-based, student centered, and community-relevant. This proposal initiated in 1983 is now supported by evidence from a diverse variety of research fields and perspectives.

*To qualify as an intelligence, each set of abilities has to fair reasonably well in meeting eight criteria as specified in *Frames of Mind* [2] (pp. 62–67):

1. Identifiable cerebral systems;
2. Evolutionary history and plausibility;
3. Core set of operations;
4. Meaning encoded in a symbol system;
5. A distinct developmental history & mastery;
6. Savants, prodigies, and exceptional people;
7. Evidence from experimental psychology;
8. Psychometric findings.

Definition: Intelligence is a biopsychological potential to process information that can be activated in a cultural setting to solve problems or create products that are of value in a culture. *Intelligence Reframed* [20] (p. 34).

## Figures and Tables

**Table 1:** Multiple intelligences core cognitive units and sample neural correlates [1].

Intelligences	Core Cognitive Units	Primary Regions	Subregions
Interpersonal	Social Perception Interpersonal Understanding Social Effectiveness Leadership	Frontal Temporal Cingulate Parietal	Medial-Temporal Amygdala Dorsolateral PFC Anterior Cingulate Superior Temporal Sulcus
Intrapersonal	Self-Awareness Self-Regulation Executive Functions Self-Other Management	Frontal Cingulate Temporal Parietal Subcortical	Prefrontal-Cortex Anterior Cingulate Dorsomedial PFC Lateral Prefrontal Ventromedial
Logical-Mathematical	Mathematical Reasoning Logical Reasoning	Frontal Parietal Temporal	Prefrontal Intraparietal Sulcus Inferior Parietal Lobule
Linguistic	Speech Reading Writing Multimodal Communication of Meaning	Temporal Frontal Parietal	Superior Temporal Gyrus Inferior Frontal Gyrus Broca’s Area Posterior Inferior Frontal Gyrus
Spatial	Spatial Cognition Working with Objects Visual Arts Spatial Navigation	Frontal Parietal Temporal Occipital	Premotor Cortex Motor Cortex Medial Temporal Prefrontal
Musical	Music Perception Music and Emotions Music Production	Frontal Temporal Subcortical Cerebellum	Superior Temporal Gyrus Primary Auditory Cortex Premotor Cortex Basal Ganglia Supplementary Motor
Kinesthetic	Body Awareness/Control Whole Body Movement Dexterity Symbolic Movement	Frontal Parietal Subcortical Cerebellum	Motor Cortex Primary Motor Cortex Premotor Cortex Basal Ganglia
Naturalist	Pattern Cognition Understanding Living Entities Understanding Animals Understanding Plant Life Science	Temporal Subcortical	Superior Temporal Sulcus Amygdala Brainstem Thalamus Midbrain Basal Ganglia

Note: The neural regions noted for each intelligence are those with the highest number of citations and are not the full list of citations in the literature. Intelligence is a complex idea that is represented by the diversity of neural structures cited for each of the multiple intelligences. See the literature [1] for full description.

**Table 2:** Five key ideas from neuroscience: Guiding a multiple intelligences-inspired education.

1. Culture Matters
2. Every Brain is Unique—Activate Strengths!
3. Know Thyself
4. Embodied Cognition and the Emotional Rudder
5. Make it Mean Something!

## Footnotes

1 For a more detailed summary see www.MIResearch.org.
